# Genomic epidemiology of antimicrobial resistance determinants in Chinese swine farm *Escherichia coli* isolates

**DOI:** 10.3389/fmicb.2025.1575426

**Published:** 2025-04-02

**Authors:** Zizhe Hu, Yee Huang, Xuemei Cui, Tuanyuan Shi, Quanan Ji, Xiangru Wang, Guolian Bao, Yan Liu

**Affiliations:** ^1^Laboratory for Bacterial Diseases of Livestock and Poultry, Institute of Animal Husbandry and Veterinary Medicine, Zhejiang Academy of Agricultural Sciences, Hangzhou, Zhejiang, China; ^2^National Key Laboratory of Agricultural Microbiology, College of Veterinary Medicine, Huazhong Agricultural University, Wuhan, China

**Keywords:** pig farms, *Escherichia coli*, antimicrobial resistance, epidemiology, whole genome sequencing, horizontal transfer of ARGs

## Abstract

**Introduction:**

The extensive use of antimicrobials in pig farming has led to a significant emergence of antimicrobial resistance (AMR) among microorganisms. Given China’s prominent position as the leading global swine producer, AMR in pig farming has become a focal point of debates. However, limited research has comprehensively assessed the overall status of AMR in Chinese pig farms.

**Methods:**

Samples were collected from 31 provinces in China. *E. coli* was identified and isolated using McConkey’s selective medium and confirmed through PCR amplification. The MIC value was determined by BD Phoenix™ M50 Automated Identification and Susceptibility device. *E. coli* J53 (sodium azide-resistant) was used as the recipient in conjugation transfer experiment. The phenotypic and genotypic characteristics of the isolates were comprehensively analyzed using whole-genome sequencing.

**Results:**

227 slurry samples were collected from 52 pig farms located across 31 provinces in seven geographical regions of China. 142 non-duplicate strains of *E. coli* were isolated, and their resistance phenotypes to 28 antimicrobials were systematically evaluated. ST10 and ST641 isolates were widespread sequence types with numerous antimicrobial resistance genes (ARGs) and virulence factor genes, including *bla*_NDM-1_, *mcr-1.1*, and *bla*_OXA-10_, among others. Multiple ARGs were co-located on a single plasmid, and an analysis of the genetic context revealed insertion sequences adjacent to ARGs containing various mobile genetic elements. Conjugation experiments provided additional evidence for the horizontal dissemination of these ARGs.

**Discussion:**

The release of *E. coli* into the environment via farm slurry comprises a significant emerging contaminant and a potential hazard to public health. Consequently, there is an urgent need to establish universally recognized farm effluent standards for monitoring the dissemination of resistant bacteria and ARGs in China’s pig farms.

## 1 Introduction

Antimicrobial resistance (AMR) is one of the most pressing global concerns and ranks among the top 10 threats to global health, as identified by the World Health Organization (WHO, 2020). A comprehensive study indicated that the estimated total number of deaths attributed to AMR in 2019 was approximately 4.95 million individuals, with drug-resistant *Escherichia coli* being responsible for the highest mortality rate (Murray et al., 2022). Further, the widespread utilization of antimicrobial agents in humans and domestic animals has led to the proliferation of antimicrobial-resistant bacteria (AMB) and the spread of antimicrobial-resistance genes (ARGs) (Mencia-Ares et al., 2020; Walsh et al., 2023). Moreover, abundant evidence suggests that livestock act as reservoirs for ARGs and AMB, particularly the microorganisms present in feces (Timmermans et al., 2021; Zhang et al., 2021). Pigs are administered the highest antibiotic amounts per kilogram of animal product among cattle, pigs, and chickens (Van Boeckel et al., 2015). China is responsible for rearing approximately half of the global pig population, and pork serves as the primary protein source in Chinese cuisine. Therefore, AMR in pig farms in China has triggered a heated discussion. Moreover, after the African swine fever epidemic, the pig-farming landscape of China has increasingly shifted toward large-scale commercial operations from the previous predominance of small-scale farms. Farmers are willing to allocate additional resources to prevent disease (Xu et al., 2022; Yan et al., 2023), and this phenomenon has led to a substantial surge in the use of antimicrobials, thereby raising concerns regarding bacterial resistance.

Microbes play an important role in ARG dissemination. The release of AMB into the environment via animal or human excrement is a well-established phenomenon (Reinthaler et al., 2003). The presence of AMB or ARGs as emerging environmental contaminants has been documented (Zhang et al., 2020). *E. coli* serves as a widely accepted biomarker or sentinel microbe for AMR surveillance (Brisola et al., 2019; Hutinel et al., 2019). It possesses a robust capacity to swiftly acquire ARGs and disseminate them to neighboring susceptible bacteria (Nolivos et al., 2019). The extensive survival of *E. coli* in diverse aquatic environments further facilitates the horizontal transfer of ARGs (Blaustein et al., 2013; Li and Zhang, 2023). Therefore, the resistance level in *E. coli* can serve as an indirect indicator of the overall antimicrobial resistance within their ecological niche. Although *E. coli* is a commensal bacterium in the gastrointestinal tract of humans and mammals, the pathogenic potential of certain serotype strains cannot be ignored, particularly in neonatal animals and infants (Denamur et al., 2020). Once these strains acquire multidrug resistance, they pose a significant threat to both livestock and human life.

Numerous studies have reported the emergence of resistant *E. coli* strains isolated from pig farms, exhibiting resistance to carbapenems, tigecycline, and polymyxins (Peng et al., 2019; Sun et al., 2019; Yuan et al., 2021; Shen et al., 2022; Wang et al., 2022). This poses a serious potential public health risk to humans. Studies published to date have extensively elucidated the prevalence of AMR in Chinese livestock through the application of metagenomic, microbiome, and retrospective analysis techniques. However, these investigations have primarily focused on specific regions and reflect local prevalence. The comprehensive status of *E. coli* AMR in pig farms across China remains to be clarified from various perspectives. Furthermore, inherent algorithmic limitations often lead to prediction errors. Geographic drug resistance monitoring exhibits higher accuracy than data prediction, and the introduction of this approach can lead to a 50% reduction in uncertainty (Zhao C. et al., 2021). Moreover, whole genome Sequencing (WGS) has proven to be effective in AMR surveillance (Gomi et al., 2024). Hence, in this study, we conducted a national surveillance by collecting slurry samples from pig farms in seven geographical regions of China and focused on ARGs harbored by commensal *E. coli*. Our investigation covered 31 provincial municipalities and autonomous regions and provided insight into the AMR profile of *E. coli* in farm slurries. The findings of this study provide valuable insights for the formulation of policies implemented to manage antimicrobial drugs in livestock.

## 2 Materials and methods

### 2.1 Sample collection and *E. coli* isolation

#### 2.1.1 Sampling

**Figure 1:**
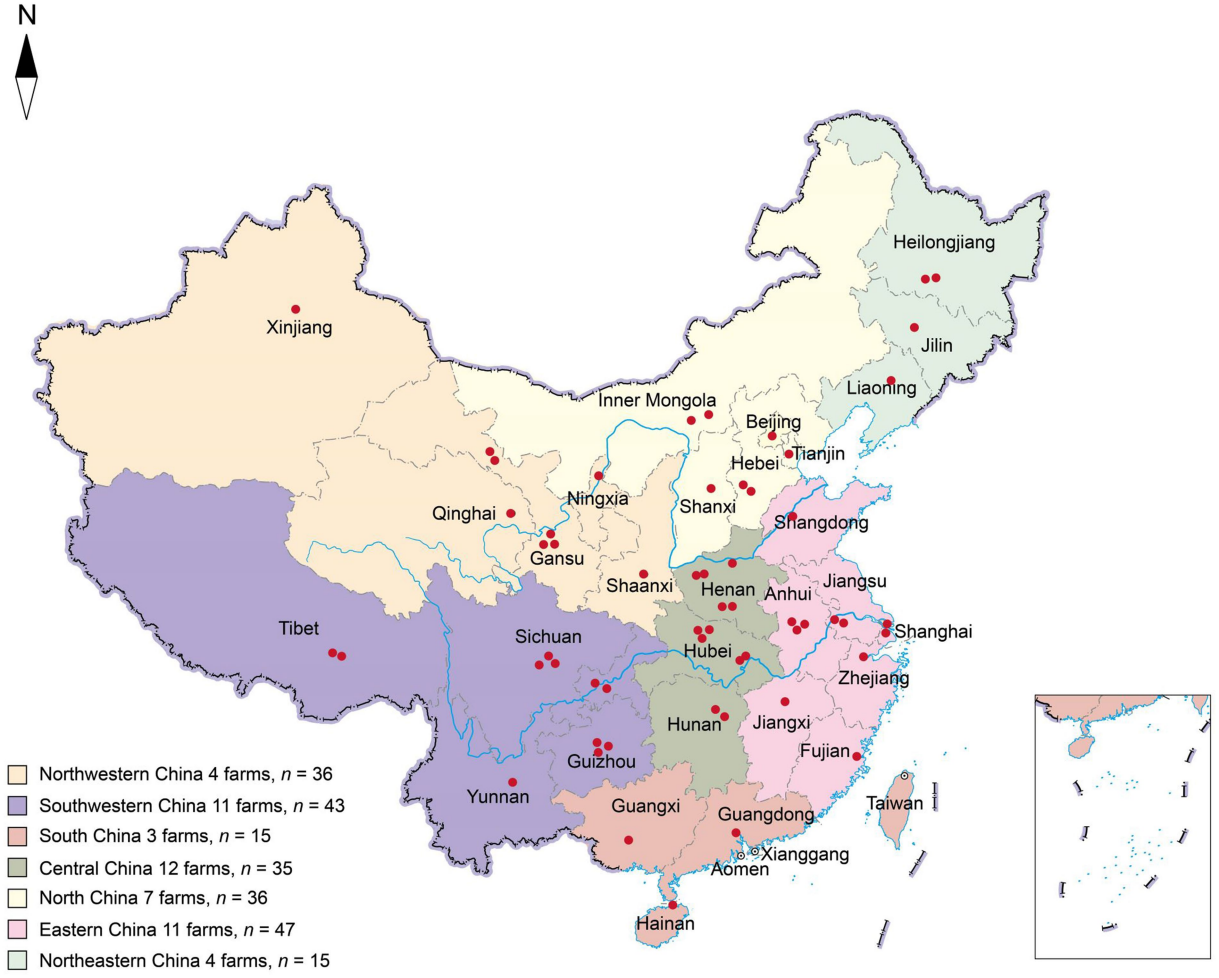
Geographical distribution of sampled pig farms across China. Each red dot represents a farm location, while the seven colors indicate the geographical divisions within China. The letter “*n*” denotes the total number of samples collected within the designated area.

Two hundred and twenty-seven fecal slurry samples were collected from 52 pig farms distributed across seven geographic districts in China, encompassing 31 provinces, between January and October, 2019. Fecal slurry samples were collected from each pigsty, based on different herds, ensuring a minimum of five samples per farm. Barn slurry samples were collected using sterile absorbent cotton swabs. Three duplicate swabs were collected and combined to form a single sample. Sterilized tubes with a 50 mL capacity were used for sample collection. The geographical locations of the farms are illustrated in Figure 1, and detailed information is provided in Table 1. The farms were located in Northwestern China (four farms, *n* = 36), Southwestern China (11 farms, *n* = 43), South China (three farms, *n* = 15), Central China (12 farms, *n* = 35), North China (seven farms, *n* = 36), Eastern China (11 farms, *n* = 47), and Northeast China (four farms, *n* = 15). All samples were promptly stored in dry ice boxes and transported via express delivery to the laboratory within 48 h.

**Table 1:** Number of samples collected and *Escherichia coli* isolation.

Source	Province	Number of samples	Total^a^	Number of isolates	Total^b^
Northeastern China	Heilongjiang	5	15	3	13
Northeastern China	Jilin	5		5	
Northeastern China	Liaoning	5		5	
North China	Inner Mongolia	5	36	1	17
North China	Hebei	10		6	
North China	Tianjin	6		1	
North China	Beijing	5		4	
North China	Shanxi	10		5	
Eastern China	Shandong	5	47	5	30
Eastern China	Shanghai	10		8	
Eastern China	Jiangsu	10		7	
Eastern China	Jiangxi	7		5	
Eastern China	Anhui	5		1	
Eastern China	Zhejiang	5		2	
Eastern China	Fujian	5		2	
Central China	Henan	15	35	9	22
Central China	Hubei	15		11	
Central China	Hunan	5		2	
South China	Guangdong	5	15	4	10
South China	Guangxi	5		4	
South China	Hainan	5		2	
Southwestern China	Tibet	10	43	9	22
Southwestern China	Yunnan	5		4	
Southwestern China	Chongqing	8		2	
Southwestern China	Guizhou	10		4	
Southwestern China	Sichuan	10		3	
Northwestern China	Qinghai	5	36	2	28
Northwestern China	Gansu	16		14	
Northwestern China	Ningxia	5		5	
Northwestern China	Shaanxi	5		5	
Northwestern China	Xinjiang	5		2	
Total^c^	–		227		142

^aTotal number of samples collected from each geographic district. bTotal number of E. coli isolates from each geographical district. cTotal number of samples and isolates.^

#### 2.1.2 *Escherichia coli* isolation, identification, and analysis of conservation

According to previous methodologies (Hu et al., 2021), the *E. coli* isolation protocol can be summarized as follows: Luria–Bertani (LB) broth (OXOID, USA) was used to preculture slurry samples; then, one loop of cultured liquid was streaked onto MacConkey agar (Haibo, China), plates were incubated in a 37°C incubator for 24 h, and single pink clones were selected for incubation on eosin–methylene blue agar (Haibo, China). Single clones with a greenish metallic sheen were selected for inoculation into LB broth, and bacterial PCR was conducted for *E. coli* identification. Reference primers (F: 5′-GAAGCTTGCTTCTTTGCT-3′, R: 5′-GAGCCCGGGGATTTCACAT-3′) were used to amplify the *E. coli* 16S rRNA sequence (Sabat et al., 2000). The purified *E. coli* was spin-coated onto LB agar and incubated at 37°C for 24 h, followed by flushing the bacterial lawn with sterilized 15% skim milk and transferring it into penicillin glass bottles. A gradient freeze dryer (Christ, Germany) was used to freeze-dry the strains, and then, the glass bottles were stored at −80°C.

### 2.2 Antimicrobial susceptibility testing (AST)

The BD Phoenix™ M50 Automated Identification and Susceptibility device (United States) was employed to assess the resistance of isolated strains to a panel of 28 antimicrobial agents, including amikacin, gentamicin, tobramycin, ertapenem, imipenem, meropenem, cefazolin, cefuroxime, cefoxitin, ceftazidime, ceftriaxone, cefepime, aztreonam, amoxicillin/clavulanate, ampicillin/sulbactam, piperacillin/tazobactam, colistin, trimethoprim/sulfamethoxazole, chloramphenicol, fosfomycin, nitrofurantoin, ciprofloxacin, levofloxacin, norfloxacin, moxifloxacin, minocycline, tetracycline, and tigecycline. According to manufacturer’s instructions, the following procedure was adopted: initially, *E. coli* isolates were cultured on LB agar, and subsequently, bacterial colonies were used to prepare 0.5 McFarland bacterial suspensions, which were then inoculated into AST broth (BD Phoenix™ AST broth, United States) with a drop of indicator (BD Phoenix™ AST indicator solution, United States) within a 15 min timeframe. Subsequently, the broth was poured into AST panels (BD Phoenix™ AST panel NMIC-413, United States), which were then loaded into the Phoenix™ M50 system for minimum inhibitory concentration (MIC) value determinations and computerized analysis. *Escherichia coli* ATCC 25922 was used as the quality control strain. The results were interpreted based on the resistance breakpoints specified by Clinical and Laboratory Standards Institute M100 29th edition and European Committee on Antimicrobial Susceptibility Testing Breakpoints v 9.0.

### 2.3 Conjugation transfer experiment

The wild-type resistant strain was employed as the donor, and *E. coli* J53 (sodium azide-resistant) was the recipient. Both the donor and recipient bacteria were initially cultured to reach the logarithmic growth phase before being combined in a 1:1 volume ratio and incubated for 10 min. Then, 80 μL of mixed bacteria was applied onto a sterile nitrocellulose membrane, while two separate nitrocellulose membranes were treated with 40 μL each of donor and recipient bacteria. Additionally, an 80 μL aliquot of sterile medium was dispensed onto a nitrocellulose film, as a blank control. The processed nitrocellulose membranes were placed on agar plates and incubated at 37°C for 12 h. After incubation, membranes were washed with PBS. Müller Hiton agar with sodium azide (100 mg/L), meropenem (10 mg/L), and fosfomycin (200 mg/L) plus 25 mg/L glucose-6-phosphate was used to screen the transconjugants and further verified by PCR amplification of *bla*_NDM-1_ and *fosA3* genes (NDM-1F: ATGGAATTGCCCAATATTAT, NDM-1R: TCAGCGCAG CTTGTC; fosA3F: CGCAAAAAATGCGCT, fosA3R: AACGCTCAGAAGCTC).

### 2.4 Whole-genome sequencing and bioinformatic analysis

#### 2.4.1 Whole-genome sequencing and annotation

In total, 142 non-duplicate isolates were isolated and subjected to whole-genome sequencing using an Illumina platform (Biomarker Technologies, Beijing, China). The bacterial genome was disrupted using the Bioruptor Pico System and purified with clean DNA beads (Vazyme, Nanjing, China). The TrueLib DNA Library Rapid Prep Kit (ExCell, Suzhou, China) was used to construct the libraries. For genome assembly, the filtered reads were assembled using Spades v3.6.2. The coding genes were predicted using Prodigal v2.6.3, and the GenBlastA v1.0.4 program was used to scan the whole genome after masking the predicted functional genes. Putative candidates were then analyzed by searching for non-mature mutations and frameshift mutations using GeneWise v2.2.0. Repetitive sequences were predicted using RepeatMasker. For functional annotation, the predicted proteins were subjected to a BLAST search (*e*-value: 1e-5) based on Nr, Swiss-Prot, TrEMBL, KEGG, eggNOG, and Blast2go databases, which were used for GO annotation. Furthermore, pathogenicity and drug resistance were investigated using a BLAST search against the Comprehensive Antibiotic Research Database (CARD). The virulence factor gene (VFG) BLAST thresholds were as follows: nucleotide identity, 90%; length coverage, 90%.

To determine the structure and location of the resistance genes, we used ONT sequencing to obtain complete genome sequences from the two isolates that were positive for *bla*_NDM-1_, *bla*_OXA-10_, and *mcr-1*. For genome assembly, filtered reads were assembled using Canu v1.5 software, and circlator v1.5.5 was used to cyclize the assembled genome. The GenBlastA v1.0.4 program was used to scan the whole genome after masking the predicted functional genes. Putative candidates were then analyzed by searching for non-mature and frameshift mutations using GeneWise v2.2.0. For functional annotation, the predicted proteins were subjected to a BLAST search (*e*-value: 1.0e-5) against Nr, Swiss-Prot, TrEMBL, KEGG, eggNOG, and Blast2go databases, which were used for GO annotation.

#### 2.4.2 Bioinformatic analysis

Center of Genome Epidemiology (CGE) MLST 2.0 version 2.0.9 was used to identify sequence types (STs) and acquire MLST allele sequences. The associated allele sequences were then used to generate a FASTA file. Subsequently, evolutionary analyses were conducted using MEGA11, and evolutionary history was inferred using the neighbor-joining method. iTOL v6 was used to visualize and modify the tree. The SerotypeFinder tool was used to predict the O and H serotypes of *E. coli* using a threshold selection of 95% and a minimum length requirement of 60%. The Clermon Typing 23.06 tool was employed to predict the phylogroups of strains. PlasmidFinder 2.1 was used to predict isolated plasmid replicons.

### 2.5 Data analysis and visualization

RAW Graphs 2.0 was used to portray the Sankey diagram. The Blast Ring image Generator v0.95 was used to visualize the plasmid blast maps in this study. Grape tree software was used to depict minimal spanning tree. Snap gene 3.3.1 was used to display the genetic structure of the ARGs.

## 3 Results

### 3.1 Resistance phenotype

**Figure 2:**
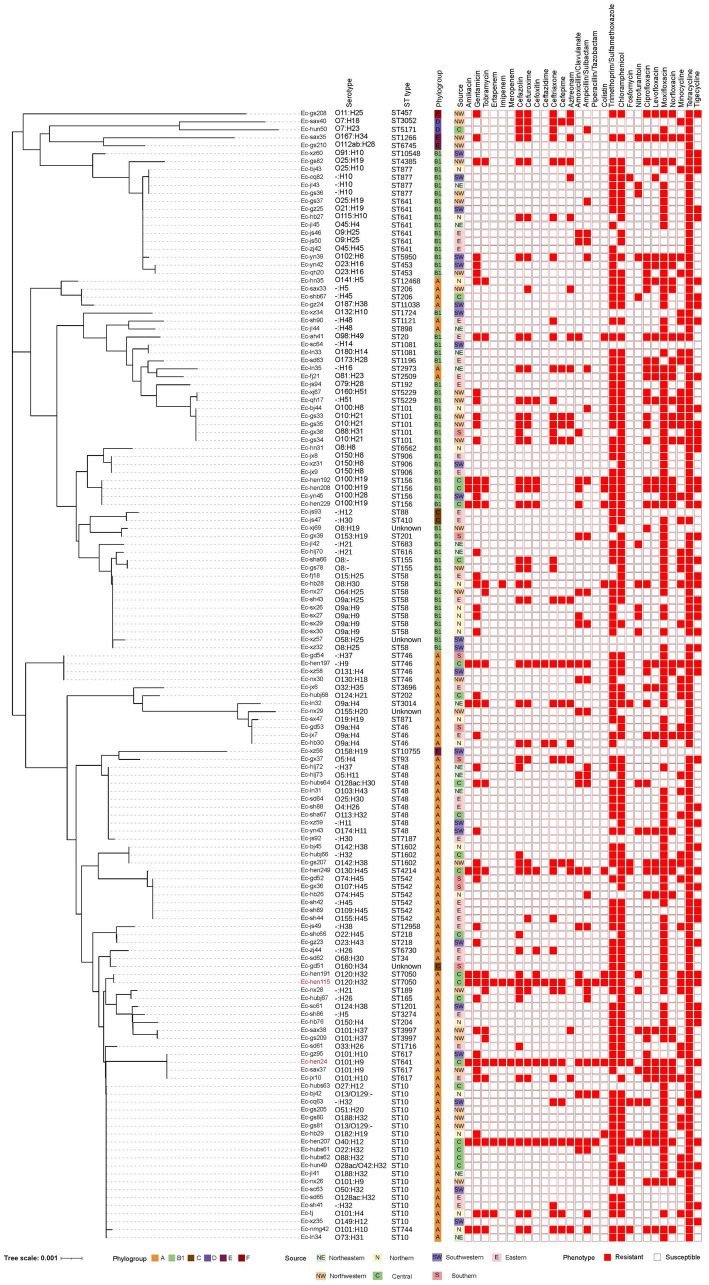
Phylogenetic relatedness, serotype, sequence type, phylogroup, origin, and antimicrobial-resistant phenotype of 142 *Escherichia coli* strains. The phylogenetic tree, constructed using neighbor-joining analysis of 142 *E. coli* housekeeping genes allele sequences, features phylogroups depicted by six distinct color squares. Isolate sources are shown by seven different color squares, while 28 antimicrobial resistance phenotypes are represented with solid graphics for resistance and hollow graphics for susceptibility.

The AMR profiles of the 142 *E. coli* strains are shown in Figure 2. Resistance rates were 33.10% (47/142), 15.49% (22/142), and 7.75% (11/142) for gentamicin, tobramycin, and amikacin, respectively. An analysis of cephalosporins, ranging from the first to the fourth generation, revealed that cefazolin was associated with the highest resistance rate at 28.87% (41/142), followed by cefuroxime and ceftriaxone, both at 24.65% (35/142). The rate of resistance to cefepime was 12.68% (18/142), whereas the rates for cefoxitin and ceftazidime were lower, at 7.75% (11/142) and 3.52% (5/142), respectively. The rates of resistance to amoxicillin-clavulanate, ampicillin-sulbactam, piperacillin-tazobactam, and aztreonam were 16.90% (24/142), 20.42% (29/142), 2.82% (4/142), and 16.20% (23/142), respectively. Furthermore, 5.63% (8/142) of the isolates displayed resistance to fosfomycin, whereas two of these isolates also demonstrated resistance to carbapenems. Notable resistance to nitrofurantoin was observed in 12.68% (18/142) of the isolates. The rates of resistance to fluoroquinolones were 30.28% (43/142), 21.13% (30/142), 23.94% (34/142), and 80.99% (115/142), for ciprofloxacin, levofloxacin, norfloxacin, and moxifloxacin, respectively. Tetracycline, trimethoprim/sulfamethoxazole, and chloramphenicol were associated with high resistance rates, specifically 96.48% (137/142), 78.17% (111/142), and 80.99% (115/142), respectively. Furthermore, 10 isolates were resistant to colistin (7.04%, 10/142), three of which also exhibited resistance to carbapenems. Resistance to the carbapenems ertapenem, imipenem, and meropenem was identified in 2.82% (4/142), 2.82% (4/142), and 2.11% (3/142) of isolates, respectively. Moreover, 34.51% (49/142) and 36.62% (52/142) of isolates were resistant to minocycline and tigecycline, respectively. The specific resistance rates and MIC values are shown in Supplementary Figure S1 and Supplementary Table S1.

### 3.2 Multilocus sequence typing, phylogroups, and serotype analysis

**Figure 3:**
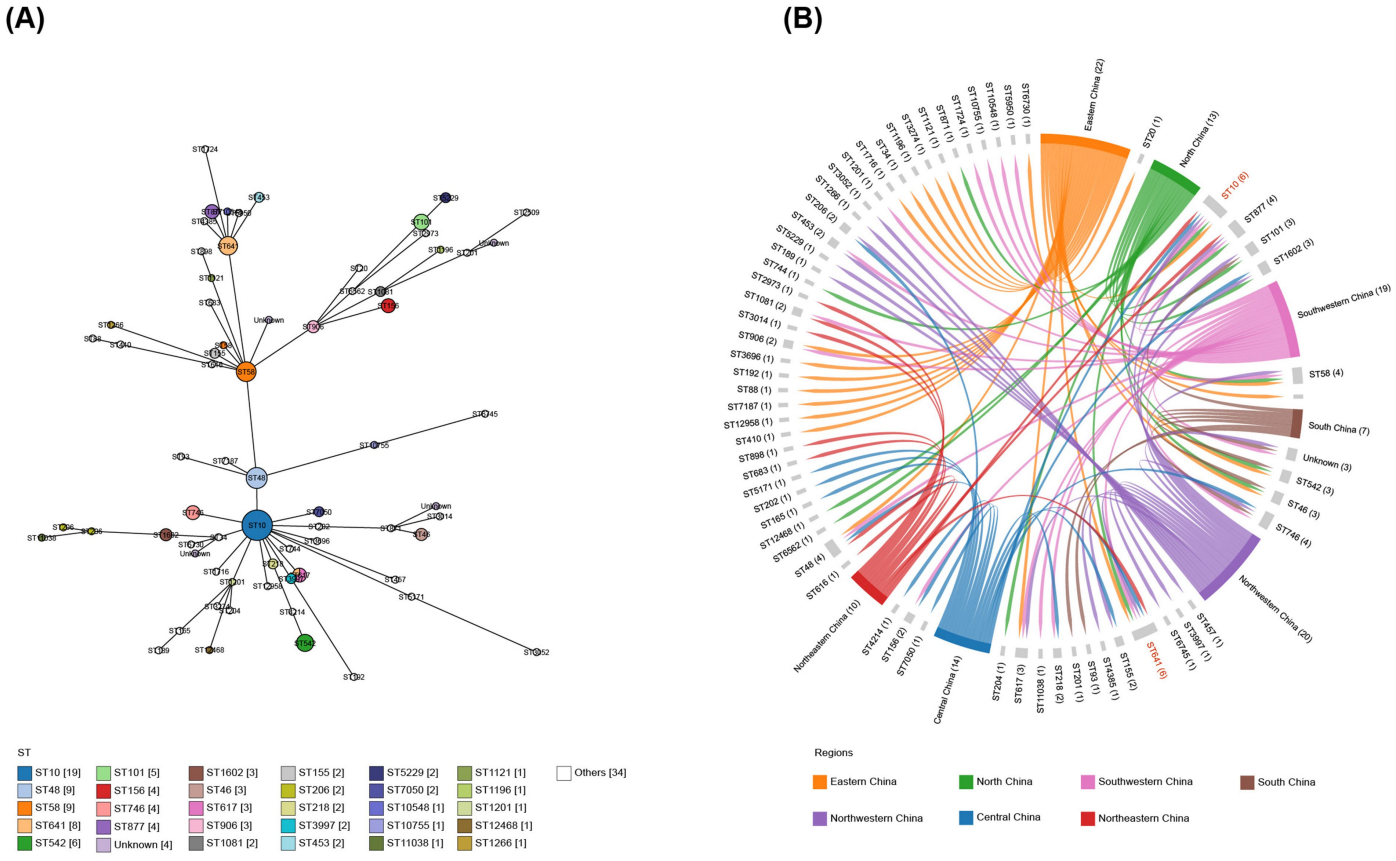
Minimum spanning tree of *E. coli* isolates and their distribution across seven geographical regions in China. **(A)** Minimum spanning tree analysis of 142 sequenced *E. coli* isolates based on sequence types. **(B)** Correlation between sequence types and the seven geographical regions, highlighting the presence of ST10 and ST641 in six of these regions.

**Figure 4:**
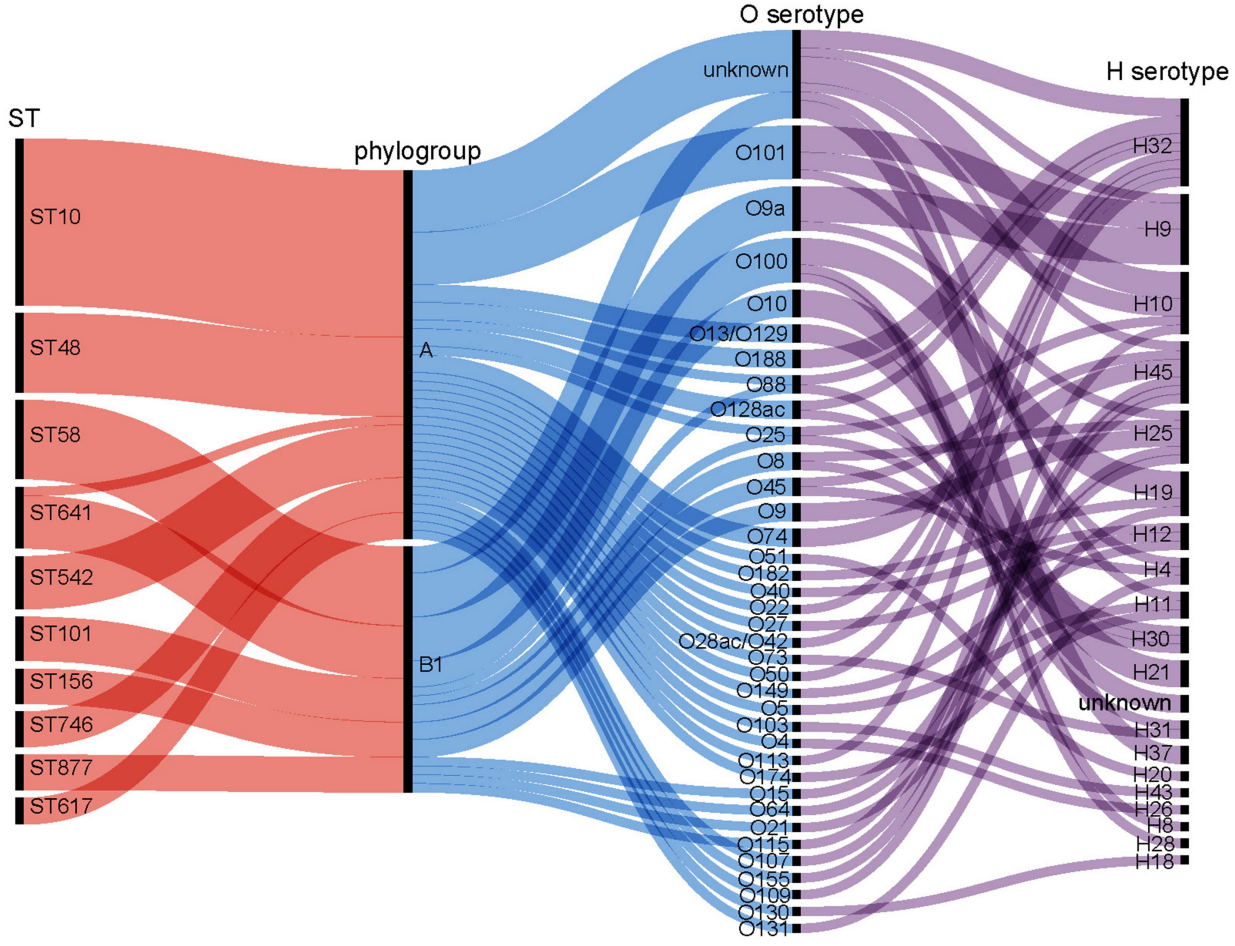
Correlation between prevalent sequence types (STs), phylogroups, O-serogroups, and H-serogroups. The isolates demonstrate diversity in O: H serotypes. STs within the A phylogroup include ST10, ST48, ST542, ST746, and ST617. The B1 phylogroup includes ST58, ST101, ST156, and ST877.

In total, 138 isolates were assigned to 63 distinct STs. The remaining four strains were of unknown STs. The allelic profiles of the seven housekeeping genes are presented in Supplementary Table S2. Based on these gene sequences, we constructed a phylogenetic tree as illustrated in Figure 2. Among these isolates, 50% were classified into 10 STs, namely ST10, ST48, ST58, ST641, ST542, ST101, ST156, ST746, ST877, and ST617. The minimum spanning tree is depicted in Figure 3A, revealing that the most prevalent clone was ST10 (19/142, 13.38%), followed by ST48 (9/142, 6.34%), ST58 (9/142, 6.34%), and ST641 (8/142, 5.63%). The distribution of strains belonging to different STs across the seven geographical regions of China is illustrated in Figure 3B. ST10 and ST641 exhibited ubiquitous distribution and were detected in all six regions, except southern China. Additionally, among the 142 strains, six phylogroups (A, B1, C, D, E, and F) were identified and a detailed BLAST result is presented in Supplementary Table S3. Phylogroup A was the most prevalent (80/142, 56.34%), followed by B1 (53/142, 37.32%). Furthermore, the predicted results of *E. coli* serotypes are presented in Supplementary Table S4. A total of 114 isolates were identified, encompassing 63 distinct O serogroups, while the remaining 28 isolates exhibited no correspondence. Among these, O101, O9a, and O8 were dominant O serogroups. In addition, 138 strains were assigned to 30 H serogroups; the dominant serogroups were H32, H15, H10, and H45, whereas the remaining four isolates did not exhibit any matches. However, the combined O and H serogroup isolates were diverse. The relationships among the prevalent STs, phylogroups, O serogroups, and H serogroups are illustrated in Figure 4. All ST10, ST48, ST542, ST746, and ST617 isolates belonged to phylogroup A, and ST58, ST101, ST156, and ST877 isolates belonged to phylogroup B1.

### 3.3 Plasmid replicon types, ARGs, and VFG analysis

The analysis of 135 isolates revealed the presence of 31 plasmid replicon types across multiple categories, while the remaining seven isolates did not exhibit any identifiable replicon types. Among them, IncFIB (*n* = 108), IncX1 (*n* = 78), IncFII (*n* = 44), and IncFIA (*n* = 40) were the most prevalent, accounting for more than 50% of these (Supplementary Figure S2). The detailed BLAST information is presented in Supplementary Table S5. The CARD database annotation yielded 156 disparate ARGs, and a comprehensive overview of all identified ARGs is provided in Supplementary Table S6. The average number of ARGs per isolate was 61.05, with a 95% confidence interval ranging from 60.00 to 62.10. Notably, most of these consisted of antibiotic efflux resistance genes. The presence of carbapenem-resistance genes (*bla*_NDM-1_, *bla*_OXA-1_, *bla*_OXA-10_, and *bla*_OXA-181_), ESBL resistance genes (*bla*_TEM_, and *bla*_CTX-M_), polymyxin-resistance gene (*mcr-1*), plasmid-mediated quinolone-resistance genes (*qnrS1*, *oqxA*, *oqxB*, and *qnrB4*), and aminoglycoside-resistance genes (*aadA2*, *aadA*, *aph(6)-Id, aph (3″)-Ib,* and *aac(3)-IIa*) are of significant concern (Supplementary Table S7). The VFDB database annotation predicted a total of 173 distinct VFGs and detailed results are shown in Supplementary Table S8. The average number of VFGs per isolate was 48.56, with a 95% confidence interval ranging from 46.40 to 50.72 (Supplementary Table S6).

### 3.4 Plasmid homology BLAST search and ARGs obtained

**Figure 5:**
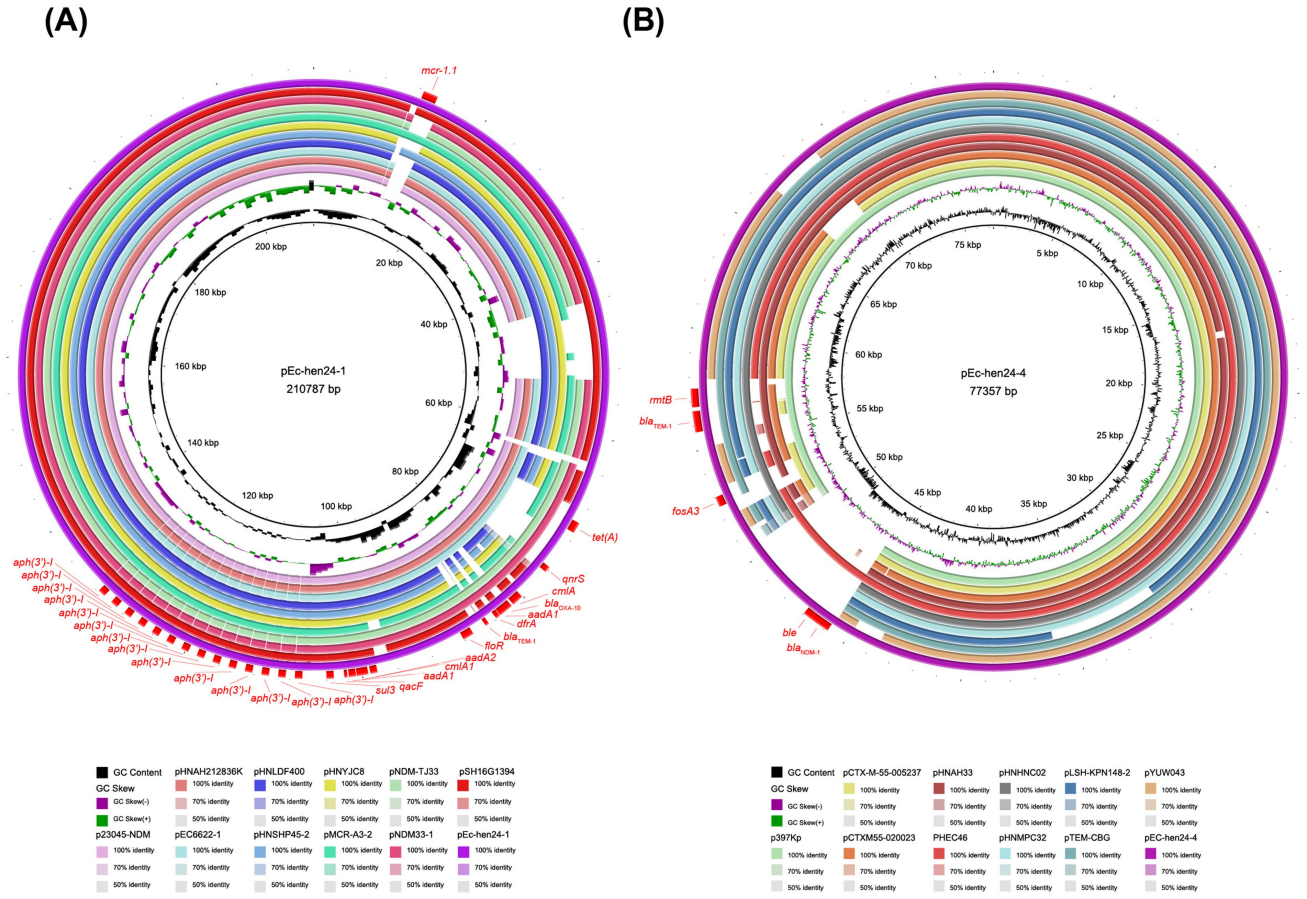
The comparison of pEc-hen24-1 and pEc-hen24-4 with their respective homologous counterparts. **(A)** The rings, from inside to outside, represent GC content, GC skew, p23045-NDM (accession no. OR497833), pHNAH212836K (accession no. CP104628), pEC6622-1 (accession no. CP096588), pHNLDF400 (accession no. KY019258), pHNSHP45-2 (accession no. KU341381), pHNYJC8 (accession no. KY019259), pMCR-A3-2 (accession no. CP059836), pNDM-TJ33 (accession no. MN915010), pNDM33-1 (accession no. MN915011), pSH16G1394 (accession no. MK477614), pEc-hen24-1 (accession no. CP155686) and resistance genes, respectively. **(B)** The rings, from inside to outside, represent GC content, GC skew, p397Kp (accession no. LN897474.2), pCTX-M-55-005237 (accession no. CP026576.2), pCTX-M-55-020023 (accession no. CP025949.1), pHNAH33 (accession no. MG197496.1), PHEC46 (accession no. KX503323.1), pHNHNC02 (accession no. MG197497.1), pHNMPC32 (accession no. MG197499.1), pLSH-KPN148-2 (accession no. CP040124.1), pTEM-CBG (accession no. CP046117.1), pYUW043 (accession no. MK778454.1), pEC-hen24-4 (accession no. CP155689) and resistance genes.

We focused on two isolates (Ec-hen24 and Ec-hen115) that displayed concurrent resistance to carbapenems, colistin, fosfomycin, and other antimicrobials. These two complete genomes were obtained using ONT sequencing. Six plasmids were assembled in Ec-hen24, including pEc-hen24-1 (GenBank accession no. CP155686), pEc-hen24-2 (GenBank accession no. CP155687), pEc-hen24-3 (GenBank accession no. CP155688), pEc-hen24-4 (GenBank accession no. CP155689), pEc-hen24-5 (GenBank accession no. CP155690), and pEc-hen24-6 (GenBank accession no. CP155691). pEc-hen24-1 harbored *mcr-1.1*, *tetA*, *qnrS1*, *cmlA1*, *bla*_OXA-10_, *aadA1*, *dfrA14*, *floR*, *aadA2*, and *sul3* ARGs. Notably, it harbored 14 copies of *aph(3′)-I*. It showed homology with plasmids in *E. coli*, *Salmonella typhimurium*, and *Klebsiella pneumonia* isolated from diarrheal outpatients, broiler farms, and poultry in China, as illustrated in Figure 5A. The pEc-hen24-4 carried *bla*_NDM-1_, *bla*_TEM-1_, *fosA3*, and *rmtB*. It exhibited homology with plasmids in *K. pneumonia*, *E. coli*, and *Salmonella enterica* isolated from humans, pork, and chicken in China, as depicted in Figure 5B. The Ec-hen115 genome also contained six plasmids: pEc-hen115-1 (GenBank accession no. CP155743), pEc-hen115-2 (GenBank accession no. CP155744), pEc-hen115-3 (GenBank accession no. CP155745), pEc-hen115-4 (GenBank accession no. CP155746), pEc-hen115-5 (GenBank accession no. CP155747), and pEc-hen115-6 (GenBank accession no. CP155748). Among them, pEc-hen115-6 harbored the *mcr-1.1* gene, and it showed homology with plasmids found in *E. coli*, *K. pneumoniae*, and *S. typhimurium* isolated from Chinese hospitals (Supplementary Figure S3A). Further, pEc-hen115-3 carried *bla*_NDM-1_, *aph(3′)-VI*, *bla*_TEM-1B_, *rmtB*, and *fosA3* and was homologous to plasmids hosted by *E. coli* and *Citrobacter werkmanii*, as depicted in Supplementary Figure S3B. The other plasmids, pEc-hen115-2 and pEc-hen115-4, harbored several ARGs, including *oqxA*, *oqxB*, *qnrS*, *dfrA12*, *aadA2*, *cmlA1*, *aadA1*, *qacF*, *sul3*, *mef(B)*, *tet(M)*, and *tet(A)*. Detailed information is provided in Supplementary Figure S4.

### 3.5 Genetic context and horizontal transfer of ARGs

**Figure 6:**
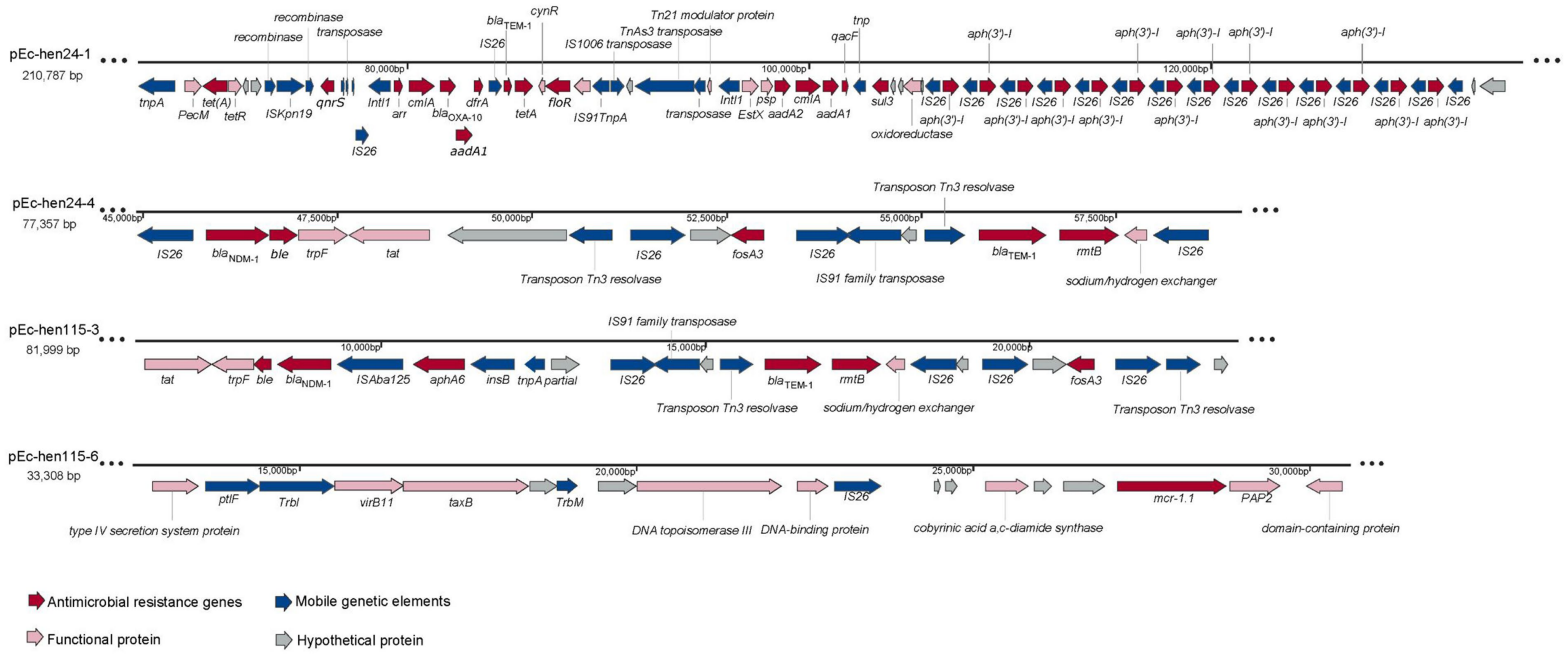
The genetic structures of antimicrobial resistance genes (ARGs) on plasmids. The presence of ARGs is represented by red arrows, mobile genetic elements are indicated by blue arrows, functional protein genes are denoted by pink arrows, and hypothetical protein genes are represented by gray arrows.

Based on genome annotation results, we conducted a genetic environment analysis of ARGs on the plasmids, as depicted in Figure 6. The plasmid pEc-hen24-1 harbored the highest number of ARGs among the 12 assembled plasmids. Notably, it concomitantly harbored *mcr-1.1*, *bla*_OXA-10_, *qnrS*, *aadA1*, *sul3*, *tetA*, *floR*, *aph(3′)-I*, and other ARGs. Additionally, in close proximity to ARGs, several mobile genetic elements, including *tnpA*, *intl1*, and IS*26*, were identified, contributing to the formation of the “*Intl1-arr-cmlA-bla*_OXA-10_*-aadA1-dfrA-*IS*26*” structure. Significantly, the *aph(3′)-I* gene was identified in close proximity to IS*26*, forming an “*aph(3′)-I-*IS*26*” arrangement with 14 repeated copies. The plasmid pEc-hen24-4 harbored *bla*_NDM-1_, *ble*, *fosA3*, *bla*_TEM-1_, and *rmtB*. The *bla*_NDM-1_ and *ble* genes were flanked by IS*26* and *trpF* (*trpF*-*bla*_NDM-1_-*ble*-IS*26*); *fosA3* was flanked by two IS*26* and a hypothetical protein open reading frame (ORF), namely “IS*26*-*ORF*-*fosA3*-IS*26*.” *bla*_TEM-1_ was connected to *rmtB*, and they were located between transposon Tn3 resolvase and a sodium/hydrogen exchanger. The pEc-hen115-3 plasmid exhibited a comparable genetic context structure (*trpF*-*bla*_NDM-1_-*ble*-IS*Aba125*”). In addition, it also carried *aphA6* flanked by IS*Aba125* and *insB* (IS*Aba125-aphA6-insB*). Plasmid pEc-hen115-6 harbored only a single ARG, *mcr-1.1*, flanked by a hypothetical protein ORF and PAP2; no mobile genetic elements were connected directly. Conjugation experiments conducted with Ec-hen24 and Ec-hen115 demonstrated that they could transfer a resistant phenotype to *E. coli* J53 recipients. Transconjugants exhibited simultaneous resistance to meropenem, ertapenem, imipenem, fosfomycin, colistin, and gentamycin, as indicated by the MIC values presented in Supplementary Table S9.

## 4 Discussion

The antimicrobials tested in this study are commonly employed for anti-infection treatment. Our findings showed that these isolates are multidrug resistant, including to carbapenems, cephalosporins, tigecycline, colistin, fosfomycin, and other hospital-used antimicrobial agents. The results implied that the emergence of AMB can occur in pig farms even in the absence of medical antimicrobials usage. Remarkably, the utilization of pig manure as an organic fertilizer is widely prevalent in China (Zhang et al., 2023). The discharge of farm slurry containing multidrug-resistant *E. coli* into the environment creates the prerequisites for its transmission within human and animal communities (Shi et al., 2021). In addition, considerable attention has been paid to the emergence of resistance to carbapenem, colistin, and tigecycline (Boutzoukas et al., 2023; Luo et al., 2024). In this work, we identified three isolates (Ec-hb28, Ec-hen24, and Ec-hen115) that exhibited simultaneous resistance to these three agents (Figure 2). Ec-hen24 and Ec-hen115 showed resistance to fosfomycin, which is an effective drug for the treatment of carbapenem-resistant *Enterobacteriaceae* (CRE) infections (Kaase et al., 2014; Sojo-Dorado et al., 2022). The use of medical pharmaceuticals is strictly regulated in pig farming operations; nevertheless, the emergence of AMB raises significant concerns about their origins and development. Animal husbandry is widely acknowledged as a reservoir for antibiotic-resistant bacteria, facilitating the transmission of both AMB and ARG to humans (He et al., 2020; Tong et al., 2022). Conversely, AMB found on farms may also potentially originate from human sources, such as hospitals or urban sewage (Wang et al., 2023; Zou et al., 2023). The AST results underscore the critical necessity to implement comprehensive antibiotic stewardship strategies in both livestock farming sectors and human healthcare to mitigate their mutual impacts.

The dominant STs resembled those identified in a previous investigation conducted on pig farms in Australia (Laird et al., 2022), implying that ST10 and ST641 could be inherently prevalent in swine farming environments. Additionally, ST10 ESBL-producing *E. coli* has been identified in river water samples collected from Nepal, indicating the existence of a globally recognized high-risk ST10 clone with a broader host range (Khanal et al., 2024). ST10 *E. coli* has also been documented in various settings, including hospital effluents, rivers, and community wastewater (Davidova-Gerzova et al., 2023). Therefore, ST10 clone may serve as a conduit for the dissemination of AMR from farms to the natural environment. Moreover, studies have reported that the ST641 *E. coli* harbors many significant ARGs, including *tetX4*, *mcr-1.1*, *bla*_NDM-4_, and *bla*_TEM_ (Diaconu et al., 2020; Zakaria et al., 2021). Our findings demonstrate the wide distribution of ST10 and ST641 clones across pig farms in China, underscoring the imperative for enhanced vigilance in detecting *E. coli* clones ST10 and ST641 on pig farms.

In this study, O101, O9a, and O8 were predominant serotypes, with no O157 isolates detected. These three serotypes of *E. coli* can cause pathogenic infections in animals and humans, as reported in previous studies (Beutin and Strauch, 2007; Denamur et al., 2020; He et al., 2021). Numerous studies have focused on O157 *E. coli*, whereas non-O157 *E. coli* has received comparatively less attention, despite its potential risks to both humans and animals (Withenshaw et al., 2022). In fact, we should pay more attention to commensal bacteria. More accurate AMR epidemiological data can be obtained by monitoring the resistance phenotypes of commensal bacteria, because pathogenic bacteria are often influenced by the use of short-term therapeutic antimicrobials. Phylogroups A and B1 of *E. coli* are commensal bacteria that normally inhabit the gastrointestinal tracts of humans and animals respectively (Tenaillon et al., 2010). They accounted for more than 90% in this work. Both phylogroups have been reported to carry ESBL genes, which have been isolated from either humans or hospitals (Jacquier et al., 2023; Chaisaeng et al., 2024). This finding further suggests a possible bidirectional influence between *E. coli* from the human gut and those found in pig farms. Additionally, the dominant populations of H serogroups primarily comprised H32, H15, and H10. However, a diverse range of O:H serotype combinations was observed. It is possible that the limited number of samples collected might not have been sufficient to adequately represent the predominant O:H serotype. The Africa swine fever virus pandemic has necessitated dedicated efforts to collect farm samples, which imposes a limitation on the scope of this study.

The presence of ARGs and VFGs indicated the substantial abundance of antimicrobial-resistant pathogens in pig farms, thereby posing a risk of infection for both farmers and farmed animals. Plasmids play an important role in the horizontal transfer of these genes. Previous studies have demonstrated that plasmids with IncFIB, IncX1, IncFII, and IncFIA replicons carry various ARGs (Li R. C. et al., 2021), particularly *mcr-1*(Li et al., 2016). In this study, whole-genome sequencing of Ec-hen24 and Ec-hen115 revealed the presence of *bla*_NDM-1_, *bla*_OXA-10_, *mcr-1.1*, and *fosA* on plasmids. These findings underscore the potential of replicon-type plasmids to preferentially harbor specific antibiotic-resistance determinants. Furthermore, these ARGs are co-localized on a single plasmid, thereby facilitating their horizontal dissemination under favorable circumstances.

There is a paucity of research on *E. coli* that is co-resistant to both carbapenem and fosfomycin in pig farms. Fosfomycin is a therapeutic agent that is effective against urinary tract infections caused by multidrug-resistant *E. coli* (Sojo-Dorado et al., 2022). Our findings provide insights into the resistance exhibited by *E. coli* present in a pig farm slurry. Carbapenem resistance genes have been identified in pig farms, leading a conclude that animal husbandry serves as a significant reservoir for CRE (Shi et al., 2021). However, the application of these drugs in pig farming is either limited or prohibited in China. The origin of these resistant strains needs to be discussed. Studies have confirmed that pharmaceutical factory sewage, hospital effluents, and suburban wastewater serve as significant reservoirs of ARGs, including CRE (Azuma et al., 2022; Khanal et al., 2024). The *bla*_NDM_-positive bacteria demonstrated persistent colonization in farms once contamination occurred through various routes (Zhai et al., 2020). Although disinfection measures can effectively eliminate viable microorganisms from objects, the persistence of microbial genomes and plasmids remains a significant concern. This persistence provides susceptible microorganisms with opportunities to acquire resistance elements, potentially leading to the transformation into antibiotic-resistant bacteria.

The analysis of genetic contexts of ARGs revealed direct associations between mobile genetic elements and ARGs. Furthermore, conjugation experiments provided additional evidence of the horizontal dissemination of ARGs. The co-occurrence of IS*26* is frequently observed with *bla*_NDM-1_, *aph(3′)-I*, *fosA3*, and other ARGs. Previous research has shown the significant role of IS*26* in facilitating the transmission of *bla*_NDM-1_ among poultry-derived *E. coli* (Zhao Q. Y. et al., 2021). Our findings further corroborate its occurrence in pig-derived *E. coli*. In addition, the “IS*26*-*ORF*-*fosA3*-IS*26*” structure formed a compound transposon, which typically consists of two insertion sequences arranged in either direct or inverted orientation, flanking a diverse array of passenger genes (Ross et al., 2021). Significant attention has been devoted to plasmid-mediated resistance gene transfer (Li and Zhang, 2023). However, the crucial roles of transposons as key facilitators of gene exchange between chromosomal elements and as important mechanisms for genetic recombination are often underestimated. The release of these ARGs and transposons into the environment through animal feces comprises a significant emerging contaminant, thereby introducing potential hazards to public health. However, to the best of our knowledge, there is no universally recognized standard for monitoring emerging pollution caused by ARGs or transposons. Currently, numerous scientific methods are available for the removal of ARGs and transposons from wastewater (Li S. et al., 2021; Gao et al., 2022; Zhong et al., 2023); however, their practical application and widespread adoption remain suboptimal.

Certain limitations were encountered in this study due to the African swine fever epidemic, as several farms declined to provide samples for biosafety considerations, resulting in a restricted sample size. Moreover, the absence of sample backup and metagenomic sequencing analysis precluded an accurate estimation of the total abundance of antibiotic ARGs present in pig farm slurry. However, we believe that ARGs harbored by live microbes may exert a more significant impact. Unfortunately, it was not feasible to collect samples from all farms workers to investigate the direct correlation between *E. coli* in the pig farm environment and the intestinal microbiota of workers. Nonetheless, our study comprehensively examined the prevalence of *E. coli* resistance in the pig farm slurry in China and provides valuable insights for future veterinary antibiotic control measures and addressing emerging pollutants in pig farms. These findings underscore the urgent need for robust antibiotic stewardship programs and surveillance systems to mitigate bidirectional AMR transmission between animal husbandry and human populations in China.

## Data Availability

All assembly whole genome sequences including plasmid sequences have been submitted to National Center for Biotechnology Information under BioProject: PRJNA1108026. Data will be made available on request.
