# Supplementary material for: Genomic epidemiology of antimicrobial resistance determinants in Chinese swine farm Escherichia coli isolates
Source: Front Microbiol. 2025 Apr 2;16:1575426. doi: 10.3389/fmicb.2025.1575426 (PMC12000052; doi:10.3389/fmicb.2025.1575426)
Supplement: Supplementary file 10 [file Data_Sheet_1.PDF]

Supplementary Material

1 Supplementary Figures and Tables

1.1 Supplementary Figures

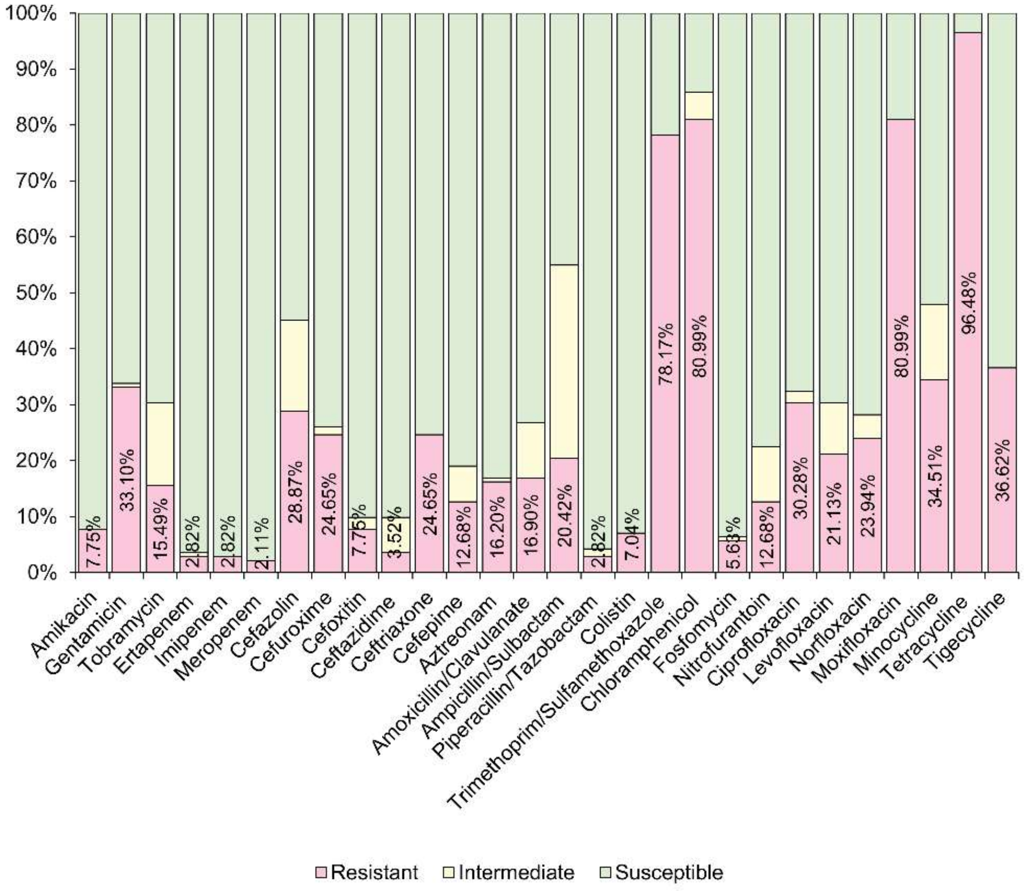

Supplementary Figure 1. The column chart displays the distribution of minimum inhibitory concentration (MIC) values for each tested antimicrobial on all isolates. The pink column represents resistance, the yellow column represents intermediate, and the green column represents susceptibility.

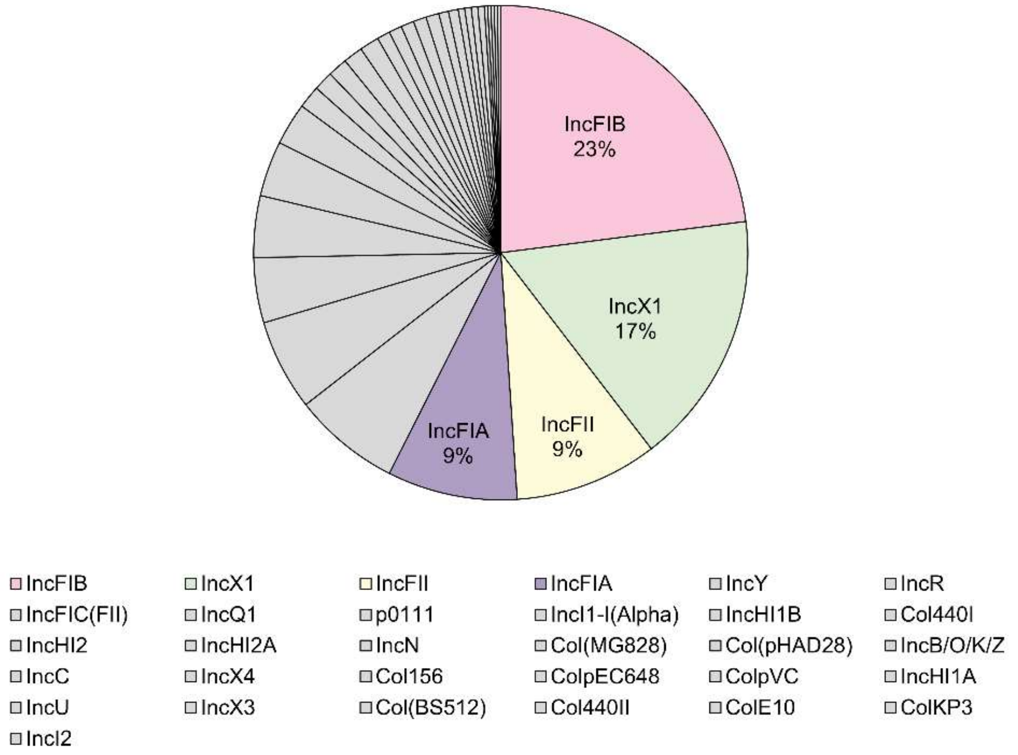

Supplementary Figure 2. Plasmid replicons identified in sequenced isolates. Among them, IncFIB (*n* = 108), IncX1 (*n* = 78), IncFII (*n* = 44), and IncFIA (*n* = 40) were the most prevalent types, accounting for more than 50%.

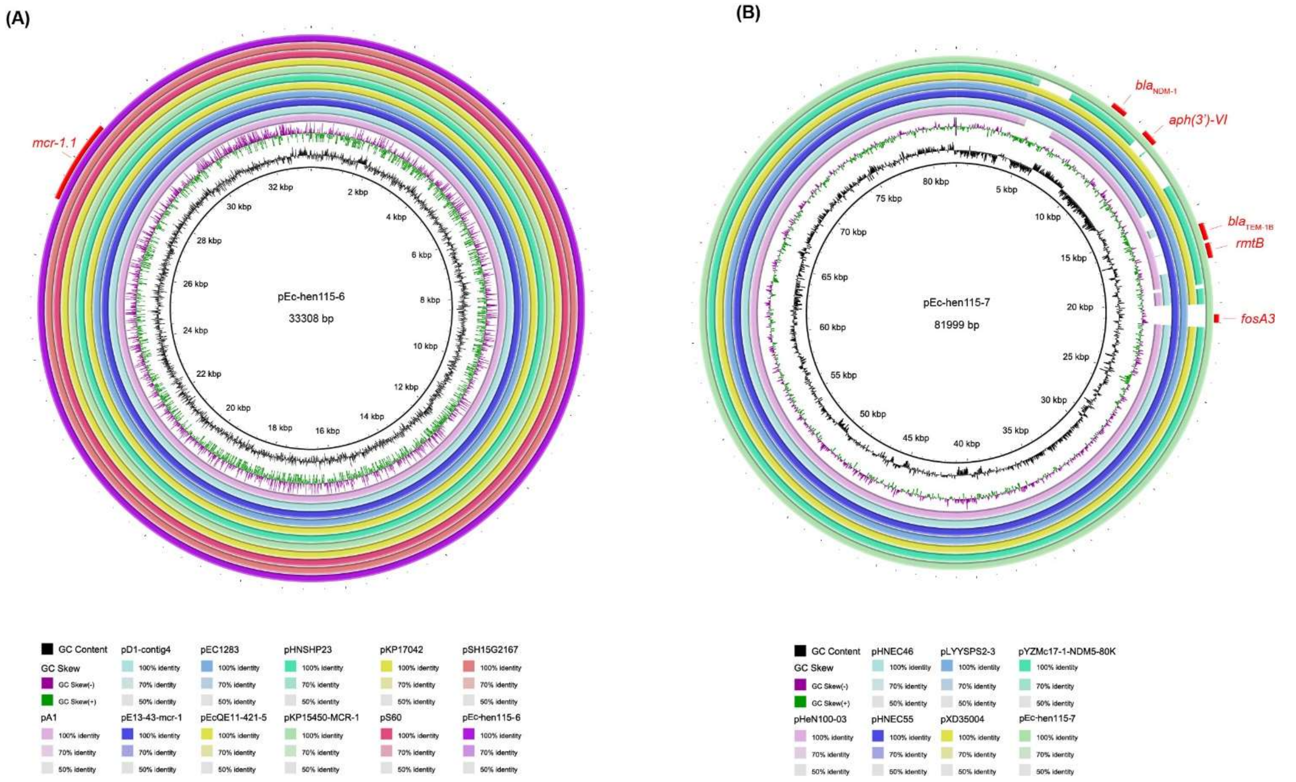

Supplementary Figure 3. Comparative analysis of pEc-hen115-3 and pEc-hen115-6 with their homologous counterparts. A. The rings, from inside to outside, represent GC content, GC skew, pA1 (accession no. LC477138.1), pD1-contig4 (accession no. MW082015.1), pE13-43-mcr-1 (accession no. MG747473.1), pEC1283 (accession no. MW010027.1), pEcQE11-421-5 (accession no. CP048826.1), pHNSHP23 (accession no. MF774184.1), pKP15450-MCR-1 (accession no. MH715959.1), pKP17042 (accession no. OL504740.1), pS60 (accession no. MW264507.1), pSH15G2167 (accession no. MK477610.1), pEc-hen115-6 (accession no. CP155748) and resistance genes respectively. **B.** The rings, from inside to outside, represent GC content, GC skew, pHeN100-03 (accession no. CP044440.1), pHNEC46 (accession no. KX503323.1), pHNEC55 (accession no. KT879914.1), pLYYSPS2-3 (accession no. MZ342958.1), pXD35004 (accession no. CP089137.1), pYZMc17-1-NDM5-80K (accession no. CP123257.1), pEc-hen115-3 (accession no. CP155745) and resistance genes.

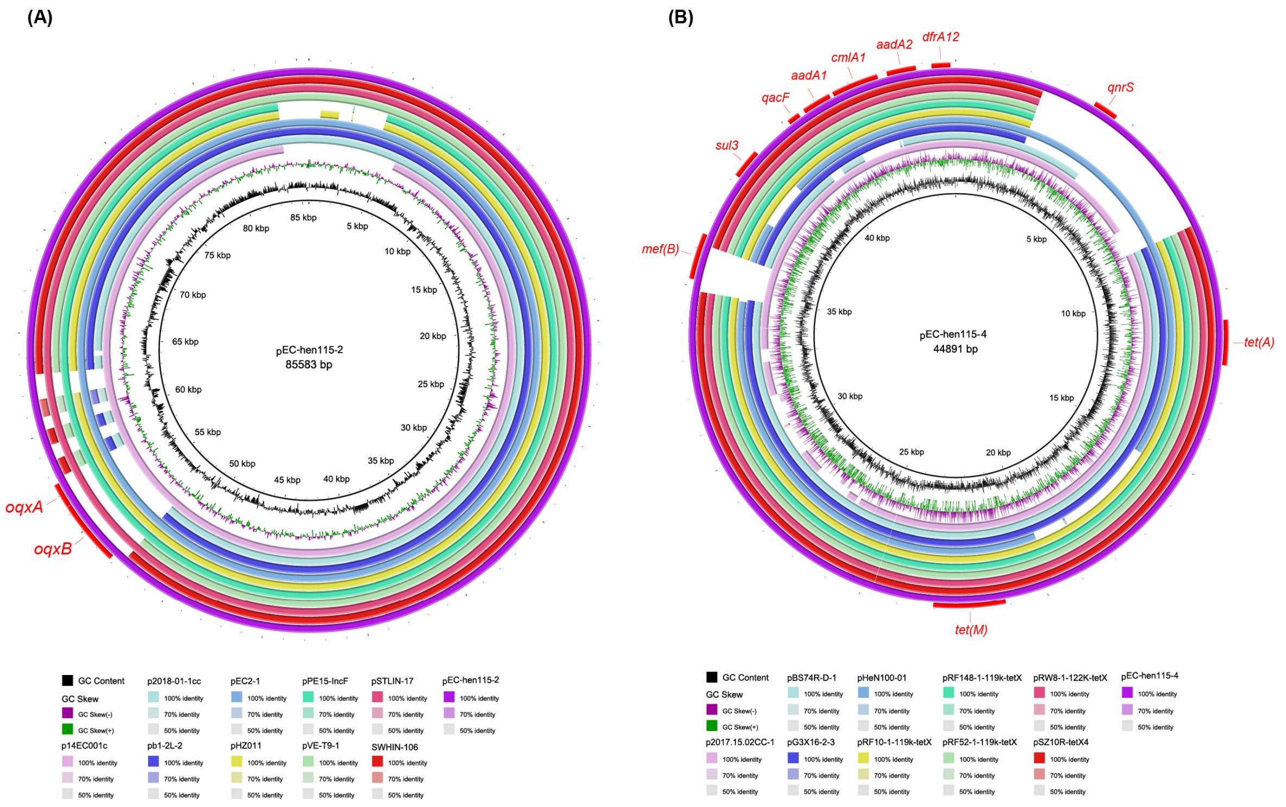

Supplementary Figure 4. The comparison of pEc-hen115-2 and pEc-hen115-4 with their respective homologous counterparts. A. GC content, GC skew, p14EC001c (accession no. CP024130.1), p2018-01-1cc (accession no. AP027870.1), pb1-2L-2 (accession no. CP072458.1), pEC2-1 (accession no. MT559985.1), pHZ011 (accession no. MN476097.1), pPE15-IncF (accession no. CP041629.1), pVE-T9-1 (accession no. AP027964.1), pSTLIN-17 (accession no. CP058781.1), SWHIN-106 (accession no. CP055108.1), pEc-hen115-2 (accession no. CP155744) and resistance genes are depicted as different color rings from inside to outside, respectively. **B.** The rings, from inside to outside, represent GC content, GC skew, p2017.15.02CC-1 (accession no. AP027819.1), pBS74R-D-1 (accession no. CP063333.1), pG3X16-2-3 (accession no. CP038140.1), pHeN100-01 (accession no. CP044438.1), pRF10-1-119k-tetX (accession no. MT219823.1), pRF148-1-119k-tetX (accession no. MT219818.1), pRF52-1-119k-tetX (accession no. MT219819.1), pRW8-1-122K-tetX (accession no. MT219826.1), pSZ10R-tetX4 (accession no. MW940628.1), pEC-hen115-4 (accession no. CP155746) and resistance genes.

1.2 Supplementary Tables

TableS1. The background information and MIC value of isolated strains.

TableS2. Sequence types of 142 sequenced *E. coli* strains.

TableS3. The result of phylogroups for sequenced *E. coli* strains.

TableS4. Annotation results for serotypes of sequenced *E. coli* strains.

TableS5. The result of plasmid replicons for sequence *E. coli* strains.

TableS6. The overview of all identified ARGs and VFGs.

TableS7. Annotation results for ARGs in sequenced *E. coli* strains.

TableS8. Annotation results for VFGs in sequenced *E. coli* strains.

TableS9 The MIC value of conjugants.
